# MiR-509-3 augments the synthetic lethality of PARPi by regulating HR repair in PDX model of HGSOC

**DOI:** 10.1186/s13045-020-0844-0

**Published:** 2020-01-31

**Authors:** Chenggong Sun, Wenyu Cao, Chunping Qiu, Chengcheng Li, Samina Dongol, Zhiwei Zhang, Ruifen Dong, Kun Song, Xingsheng Yang, Qing Zhang, Beihua Kong

**Affiliations:** 1 Department of Obstetrics and Gynecology, Qilu Hospital, Shandong University, 107 West Wenhua Road, Ji’nan, Shandong 250012 People’s Republic of China; 2 Gynecology Oncology Key Laboratory, Qilu Hospital, Shandong University, Ji’nan, Shandong 250012 People’s Republic of China

**Keywords:** miR-509-3, PARPi, Synthetic lethality, RAD51, PDX

## Abstract

**Background:**

PARP inhibitors have been the most promising target drugs with widely proven benefits among ovarian cancer patients. Although platinum-response, HR-related genes, or HRD genomic scar detection are acceptably used in assessment of Olaparib response, there are still evident limitations in the present approaches. Therefore, we aim to investigate more accurate approaches to predict Olaparib sensitivity and effective synergistic treatment strategies.

**Methods:**

We probed two databases (TCGA and Qilu Hospital) in order to quest novel miRNAs associated with platinum-sensitivity or HR-related genes. Cellular experiments in vitro or in vivo and PDX models were utilized to validate their role in tumor suppression and Olaparib sensitizing. Furthermore, HR gene mutation was analyzed through WES to explore the relation between HR gene mutation and Olaparib response.

**Results:**

High miR-509-3 expression indicated better response to platinum and longer progression-free and overall survival in two independent ovarian cancer patient cohorts (high vs. low miR-509-3 expression; PFS: TCGA *P* < 0.05, Qilu *P* < 0.05; OS: TCGA *P* < 0.05, Qilu *P* < 0.01). MiR-509-3 could impair the proliferation, migration, and invasion ability but enhance the sensitivity to Olaparib of ovarian cancer cell in vitro and in vivo by directly targeting HMGA2 and RAD51. In two PDX cases (PDX1 and PDX9), miR-509-3 could significantly increase the sensitivity to Olaparib along with the decrease of RAD51 positive rate (mean tumor weight NC + Olaparib vs. miR-509 + Olaparib; PDX1 *P* < 0.05, PDX9 *P* < 0.05). Additionally, in PDX8, miR-509-3 treatment dramatically reversed the Olaparib insensitivity (*P* < 0.05) by downregulating RAD51 expression. RAD51 functional detection revealed that all Olaparib sensitive cases exhibited low RAD51 positive rate (lesser than 50%) in treated groups. Furthermore, among the four HR gene mutation patients, three harbored HR core gene mutation and were sensitive to Olaparib while the remaining one with non-HR core gene mutation did not respond well to Olaparib.

**Conclusions:**

MiR-509-3 can sensitize ovarian cancer cells to Olaparib by impeding HR, which makes it a potential target in PARPi synergistic treatment. HR core gene analysis and RAD51 functional detection are prospectively feasible in prediction of PARPi response.

## Background

Ovarian cancer is a malignant tumor that seriously threatens women’s health; and of its many subtypes, high-grade serous ovarian carcinoma (HGSOC) is the most common and lethal one. The American Cancer Society (ACS) statistics reported that the new incidence of ovarian cancer accounted for 3% of tumors among women in 2019, and its mortality rate ranking fifth [1]. Despite continuously improving living standards, surgical and chemotherapeutic techniques in the recent several decades, the overall survival rate of ovarian cancer has not improved significantly, and the 5-year survival rate is only about 30% [2, 3]. At present, the most effective adjuvant therapy for HGSOC is the platinum and paclitaxel combination-based chemotherapy. However, 70% of patients relapse after primary therapy as a result of chemotherapy insensitivity or resistance [2, 3].

DNA damage commonly occurs in cell due to exogenous and endogenous stressors, while cells also have consequently evolved a rounded DNA damage response (DDR) system which encompasses complex signal pathways to remove or tolerate DNA damage [4] such as mismatch repair (MMR), non-homologous end joining (NHEJ) and homologous recombination (HR), etc. DDR defect creates vulnerability in specific cancer cells which provides us with potentially effective anticancer therapies [5]. It is well known that homologous recombination repair (HRR) pathway alteration is important characteristics in HGSOC. In particular, approximately 35% of HGSOCs carry BRCA1 or BRCA2 (BRCA1/2) mutation and other 6–10% of cases harbor germline or somatic mutations of at least ten other genes in the HR pathway, such as ATM, CHEK1, CHEK2, etc. [6–8]. The poly (ADP­ribose) polymerase inhibitors (PARPi), a compensatory DDR pathway inhibition, have been the most promising target therapy for HGSOC based on a conception of synthetic lethality [9, 10]. The SOLO1 study showed that Olaparib, the most commonly used PARPi, could significantly benefit the patients with newly diagnosed advanced ovarian cancer and BRCA1/2 mutation by reducing the 70% risk of disease progression or death than placebo group (hazard ratio, 0.30; 95% CI 0.23 to 0.41; *P* < 0.001) [11]. Based on this result, in December 2018, the Food and Drug Administration (FDA) of USA has approved Olaparib as the first-line maintenance therapy in adult patients with advanced epithelial ovarian cancer, fallopian tube cancer, or primary peritoneal cancer who exhibited complete or partial clinical response after platinum-based chemotherapy and harbored deleterious or suspected deleterious germline or somatic BRCA mutations. Although platinum response was used to be a feasible indication for PARPi application, HR-related gene mutations (mainly BRCA1/2 mutation) remain the most convincing genetic indicators for response to PARPi. However, there were still 40–70% patients with BRCA1/2 mutations who failed to respond to PARPi [12–14]. Furthermore, HR deficiency (HRD) genomic scar detection was developed and used in guiding PARPi therapy. But the NOVA and Arial 3 study revealed that HRD negative patients could also benefit from PARPi [15, 16]. More specific and accurate methods to predict the response to PARPi and overcome the resistance warrant further investigation.

In this study, we firstly identified miRNA-509-3 as a prognostic and platinum-sensitive factor through bioinformatic analysis of miRNA data on TCGA database. Meanwhile, we established a patient-derived-xenograft (PDX) model to reveal that miR-509-3 plays an essential role in HR pathway regulation and tumor suppression and therefore increases the sensitivity to PARPi therapy in HGSOC. Furthermore, we detected the HR-related gene mutation and RAD51 expression level of each PDX parent tissue through whole-exome sequencing (WES) in order to investigate their correlation with Olaparib response.

## Materials and methods

### Bioinformatic analysis

Raw data was downloaded from TCGA website including the miRNA expression profile of each case and the corresponding clinical data. According to NCCN Guidelines for Ovarian Cancer (2019, V1), the platinum response status were judged, which divided the cohort into platinum-sensitive (P-sen) group or platinum-resistant (P-res) group. The differential analysis on miRNA expression between the two groups was performed using the DEGseq R package [17]. *P* value was adjusted using *q* value [18]. *Q* value < 0.05 and log2 fold change absolute value > 1 were set as the threshold for significantly differential expression by default.

### Tissue samples and clinical data

A total of 126 HGSOC FFPE (formalin-fixed, paraffin-embedded) samples with detailed clinical data were collected from pathology department of Qilu Hospital, Shandong University. All patients were followed up for at least 5 years. The patients were staged by FIGO Staging System (8th ed., 2017) and distinguished into P-sen and P-res groups. The complete clinical characteristic of these enrolled patients is reported in Additional file 6: Table S1. All samples were used based on the patients’ or their guardians’ informed consent. Ethical approval was obtained from the Ethics Committee of Shandong University.

### RNA extraction and real-time quantitative PCR

AllPrep DNA/RNA FFPE Kit (QIAGEN) was used to extract the total RNA (including small RNAs) from the FFPE tissue sections. As for the cultured cells, total RNA was extracted by TRIzol reagent (Ambion) following the manufacturer’s protocol. The miRNA and mRNA were reverse-transcribed using One Step PrimeScript miRNA cDNA Synthesis Kit (Takara) and PrimeScript RT Reagent Kit (Takara) respectively. The cDNA were used as templates for real-time quantitative PCR (RT-qPCR), which was performed using SYBR Green qPCR master mix (Takara).

### Cell lines and cell culture

Human ovarian cancer cell line UWB1.289 (BRCA1-null) was purchased from American Type Culture Collection (ATCC). A2780, HEY, and HEK293T cell line was obtained from the Chinese Academy of Sciences (Shanghai, China). UWB1.289 and A2780 were cultured by RPMI 1640 medium (GIBCO) with 10% fetal bovine serum (FBS). HEY and HEK293T were cultured in DMEM (GIBCO) containing 10% FBS (BIOIND). All the cell lines were maintained at 37 °C with 5% CO_2_ in a humidified incubator.

### Stable and transient transfection

Lentivirus expressing premiR-509-3 and corresponding negative control (NC) were purchased from Genechem (Shanghai, China). Further, 1 × 10^5^ cells were plated into 6-well plates 24 h before stable transfection. The lentivirus was added into the culture medium with the multiplicity of infection (MOI) value ranging from 20 to 40. After 24 h, previous medium was replaced by fresh culture medium containing 2 μg/mL puromycin (Sigma-Aldrich) once every 2 days to obtain the stably transfected multiple colonies.

The specific small interfering RNA (siRNA) and negative control siRNA were synthesized by GenePharma (Shanghai, China) with the following sequences: HMGA2-si1 5′-CGCCAACGUUCGAUUUCUATT-3′, HMGA2-si2 5′-GGAAGAACGCGGUGUGUAATT-3′. The HMGA2 cDNA (in pEnter), RAD51 cDNA, and blank pEnter vector were purchased from Vigenebio (Shandong, China). Cells were transfected with Lipofectamine 2000 reagent (Invitrogen) according to the manufacturer’s protocol and harvested after 24–48 h for the following assays.

### Cell migration and invasion assays

The cells’ ability of migration and invasion was evaluated using the transwell technique which was performed in Cell Culture Insert (24-wells, 8.0 μm pore size, FALCON) with and without Matrigel Matrix (CORNING) respectively. Then, 1–1.5 × 10^5^ cells resuspended in 200 μL FBS-free medium were seeded into the upper chambers of culture inserts and 700 μL culture medium containing 20% FBS was injected into the lower chambers as chemoattractants. The chambers were incubated at the 37 °C incubator for an appropriate time (6–24 h). Cells in the lower surface of chambers were fixed in methanol for 15 min, stained with 0.1% crystal violet for 20 min, and counted under a light microscope.

### Cell viability and clonogenic assays

To examine the proliferation ability, each kind of cell was seeded in 96-well plates at densities of (0.8–1) × 10^3^ cells per well in quintuplicate for 0–5 days. At specific time points, 20 μL 5 mg/ml MTT (3-(4, 5)-dimethylthiahiazo (-z-y1)-3,5-diphenytetrazoliumromide, Sigma-Aldrich) was added into each well and the plate was incubated in the 37 °C incubator. After 5 h, the supernatants in wells were carefully discarded and 100 μL DMSO (Sangon Biotech) was added into each well. The absorbance value at 490 nm was evaluated by Varioskan Flash microplate reader (Thermo Scientific).

For the colony formation assays, 600–800 single cells in 10%-FBS culture medium were seeded onto 6-well plates and incubated at 37 °C for 10–14 days. The resulting colonies were fixed with methanol and stained with 0.1% crystal violet. Colony formation ability was quantified by counting the surviving colonies containing more than 50 cells.

### Cell cycle assay

The transiently transfected cells with miRNA mimics or negative control (both synthesized by GenePharma) were harvested and stained with propidium iodide according to the manufacturer’s protocol (MultiSciences, China). The treated cells were analyzed using a flow cytometer (FACSCalibur, BD, USA). The results were analyzed through Modifit LT software.

### Drug resistance assay

Drug resistance assay was measured with MTT method as mentioned above. Cells were seeded onto 96-well plates (2000–3000 cells/well) and exposed to cisplatin (S1166, Selleckchem, Houston, TX, USA) or Olaparib (AZD2281, Selleckchem, Houston, TX, USA) at various final concentrations for 24–36 h (cisplatin) or 48–60 h (Olaparib). The final viability was estimated using the MTT reagent and surviving fractions were calculated.

### Luciferase reporter assay

The 3′ untranslated regions (3′UTR) of HMGA2 or RAD51, potential target genes of miR-509-3, were synthesized by GenePharma (Shanghai, China) and inserted into pmirGLO vector (Promega) at the SacI and XhoI sites as the wild-type constructs. Mutant constructs were generated by overlap extension PCR. HEK293T cells were seeded in 96-well plate (2–3 × 10^4^/well) and co-transfected with 50 ng constructs and 0.5 pmol miR-509-3 mimics or negative control with Lipofectamine 2000 reagent. After 36 h of transfection, luciferase activity was measured using the Dual-Glo Luciferase Assay System (Promega).

### Tumor formation assay in nude mice

The HEY and UWB1.289 cell lines overexpressing miR-509-3 or corresponding negative control (NC) were used in these assays. For tumor metastasis assay, 2 × 10^6^ cells resuspended in 200 μL PBS were injected into the peritoneal cavity of 4–5-week-old female athymic BALB/c nude mice (NBRI of Nanjing University, China). After 2–3 weeks, these mice were sacrificed and examined for the growth and metastasis of the tumors in peritoneal cavity.

For drug resistance assay in vivo, paired tumor cells (5 × 10^6^ cells in 150 μL PBS per site) were injected subcutaneously into opposite sides of the axilla of ten 4-week-old nude mice. Two weeks after tumor cell injection, mice were randomly separated into treated group (*n* = 5) which were treated with olaparib (50 mg/kg) intraperitoneally and untreated group (*n* = 5) with DMSO dilution injection. After treatment once a day for 2 weeks, mice were sacrificed and their tumors were harvested and photographed. Tumors were weighed to assess the growth of each group. All the animal experiments were performed with the approval of Shandong University Animal Care and Use Committee.

### Immunofluorescence assay

Cells (8 × 10^4^) were seeded onto 24-well glass bottom plate (Cellivis) and treated with irradiation (Precision X-ray Inc.) of 4 Gy to induce DNA damage. Cells were fixed with 4% paraformaldehyde for 15 min and permeabilized with 0.1% TritonX-100, blocked with normal goat serum for 30 min at room temperature, and incubated with the RAD51 primary antibody overnight at 4 °C. The next day, the cells were incubated with secondary antibody, followed by counterstaining with DAPI. The fluorescence images were captured using the Opera Phenix™ High Content Screening System (PerkinElmer).

### Western blot analysis

Cultured cells were lysed in RIPA Lysis Buffer (Beyotin, China) with 1% PMSF and 1% NaF by incubating on ice for half an hour. The supernatant were obtained by centrifugation and the protein concentration was determined by BCA Assay Kit (Thermo Scientific). A total of 30 μg protein samples per well were separated by SDS-PAGE (5.5% stacking gel and 11% separation gel), transferred to PVDF membranes (Millipore, ISEQ00010) by BIO-RAD Trans-blot (15 V, 90 min), and blocked in 5% non-fat milk solution at 25 °C for 2 h. The membrane was incubated overnight at 4 °C in the diluted primary antibody and then rinsed with TBST before incubating with the appropriate horseradish peroxidase-linked secondary antibodies for 1.5 h at 25 °C. The bands signal was detected using enhanced chemiluminescence (ECL) detection kit (PerkinElmer) by Image Quant LAS 4000 (GE Healthcare Life Sciences). β-Actin was detected as the endogenous control.

### Antibodies and reagents

Antibodies for EMT, cell cycle, and DNA damage repair pathway were purchased as follows: ZEB-1 (CST:3396), N-CAD (CST:13116), E-CAD (CST:3195), Slug (CST:9585), Snail (CST:3879), Vimentin (CST:5741), β-catenin (CST:8480), CCND1 (CST:92G2), p21 (CST:2947 s), CDK4 (Abcam: Ab108357), CDK6 (Abcam:Ab124821), total ATM (CST:2873), phospho-ATM (Ser1981) (Abcam:5883), phospho-Chk2 (Thr68) (CST:2197), phospho-H2AX (Ser139) (CST:9718), RAD51 (Abcam:ab133534), and β-actin (CST:3700). All the western blot primary antibodies were 1:1000 diluted except RAD51 (1:10000 diluted). The antibody of HMGA2 (Proteintech, 20795-1-AP) was used in Immunohistochemistry (IHC).

### PDX model establishment and therapy assay

Four- to six-week-old male NCG (NOD-Prkdc^em26Cd52^IL2rg^em26Cd22^/Gpt) mice were purchased from NBRI (Nanjing Biomedical Research Institute of Nanjing University, China). All NCG mice were housed in SPF room. The fresh primary ovarian carcinoma tissues were obtained from Department of Obstetrics and Gynecology, Qilu Hospital, Shandong University after acquiring the patients’ written informed consent. The collection of human tissue specimens and the PDX experimental procedures were approved by the Shandong University Animal Care and Use Committee.

Fresh patient tissues labeled as passage 0 (P0) were obtained from debulking surgeries in Qilu hospital within half an hour. Small tumor pieces (~ 10 × 10 × 10 mm) were washed by PBS solution and cut into homogenate. The homogenate was suspended with isometric PBS solution and mixed with Matrigel Matrix (CORNING). Then the homogenate was injected subcutaneously into the lower dorsal flank or axilla of the NCG mice. When the tumor size reached to 10 × 10 × 10 mm (often about 1–2 months later) which was labeled as P1, they were cut out and xenografted subcutaneously into another recipient mice as P2.

P2 were harvest from the sacrificed mice when tumors reached to 10 × 10 × 10 mm and made into homogenate according to aforementioned protocol. Isometric homogenate was intraperitoneally injected into new recipient mice. After 6 weeks, mice were randomly separated into three groups: NC + DMSO, NC + Olaparib, and miR-509-3 + Olaparib. The miR-509-3 groups were treated via intraperitoneal administration with adeno-associated virus (AAV, synthesized by Genechem, Shanghai) overexpressing premiR-509-3 and the other two groups accepted the AAV-NC (Genechem, Shanghai) injection. One week later, the Olaparib groups were exposed to Olaparib by intraperitoneal injection (50 mg/kg/mouse) once a day for 2 weeks while the DMSO group accept DMSO dilution injection. When treatment was completed, mice were sacrificed and their tumors harvested. Peritoneal metastasis’ weights (represent the tumor burden) and locations were recorded and compared among different groups.

### Co-immunoprecipitation

Cells were lysed in cell lysis buffer (P0013; Beyotime, Shanghai, China). For co-immunoprecipitation with cell extracts, rabbit anti-human HMGA2 polyclonal antibody (Proteintech, 20,795-1-AP), normal rabbit IgG (CST, Cell Signaling Technology) was added and incubated for 12 h at 4 °C. A mixture of equal amounts of protein G Agarose (Fast Flow, Beyotime Biotechnology) was added and incubated for another 2 h at 4 °C. Immunoprecipitates were washed five times with lysis buffer and then denatured by boiling in 1% SDS.

### Immunohistochemistry

The fresh tumor tissues were fixed by formalin for at least 24 h and cut into 4-μm-thick sections. Xylene and ethanol were used to deparaffinize and rehydrate. Antigen retrieval was achieved in 98 °C EDTA buffer (pH = 8.0) for 15 min. Endogenous peroxidase and nonspecific binding were blocked with 3% hydrogen peroxide and goat serum respectively. The primary antibody anti-HMGA2 (1:1000 diluted) and anti-RAD51 (1:500 diluted) were incubated in humid chamber overnight at 4 °C. Expression was detected by I-View 3,3′-diaminobenzidine (DAB) staining detection.

### Whole-exome sequencing and mutation annotation

Nine pairs of genomic DNA (from FFPE HGSOC tissues and corresponding normal myometrium as control) were extracted using a salting-out procedure. Whole-exome capture was performed by Sure Select Human All Exon 60 Mb kit (Agilent Technologies, Santa Clara, CA) according to the standard protocol. The captured template DNA fragments of the constructed libraries were sequenced through the Illumina HiSeqX sequencing system to generate 150-bp paired-end reads. The paired-end reads from whole-exome sequencing (WES) were first aligned to human reference genome (hg19) using BWA (version 0.7). PCR duplicates were then marked in the alignments using Picard tools (version 1.1). The somatic mutation calling was carried out by Var Scan (version v2.3.9). For each candidate somatic mutation site, the common variants in the 1000 genomes (MAF > 5%) and the intergenic or introgenic region variants were also excluded in the following analysis. The latent effects of the somatic SNP and insertion-deletion were annotated using ANNOVAR.

### Statics analysis

GraphPad Prism 6 (GraphPad Software, USA) was mainly used in data analyzing. Chi-squared test was used to analyze the differences in clinical characteristics. Survival analysis was performed by Kaplan-Meier and Log-rank test. Multivariate survival analysis was achieved by Cox regression in SPSS 17.0 (SPSS Inc., Chicago, IL). Student’s *t* test and one-way ANOVA analysis were applied to determine the statistical differences among different groups. Data are represented by means ± standard deviation of at least three independent experiments. *P* < 0.05 was considered to be statistically significant (*P < 0.05; **P < 0.01; ***P < 0.001; ****P < 0.0001).

## Result

### MiR-509-3 expression is increased in platinum-sensitive HGSOC patients and its high expression predicts better survival

**Fig. 1:**
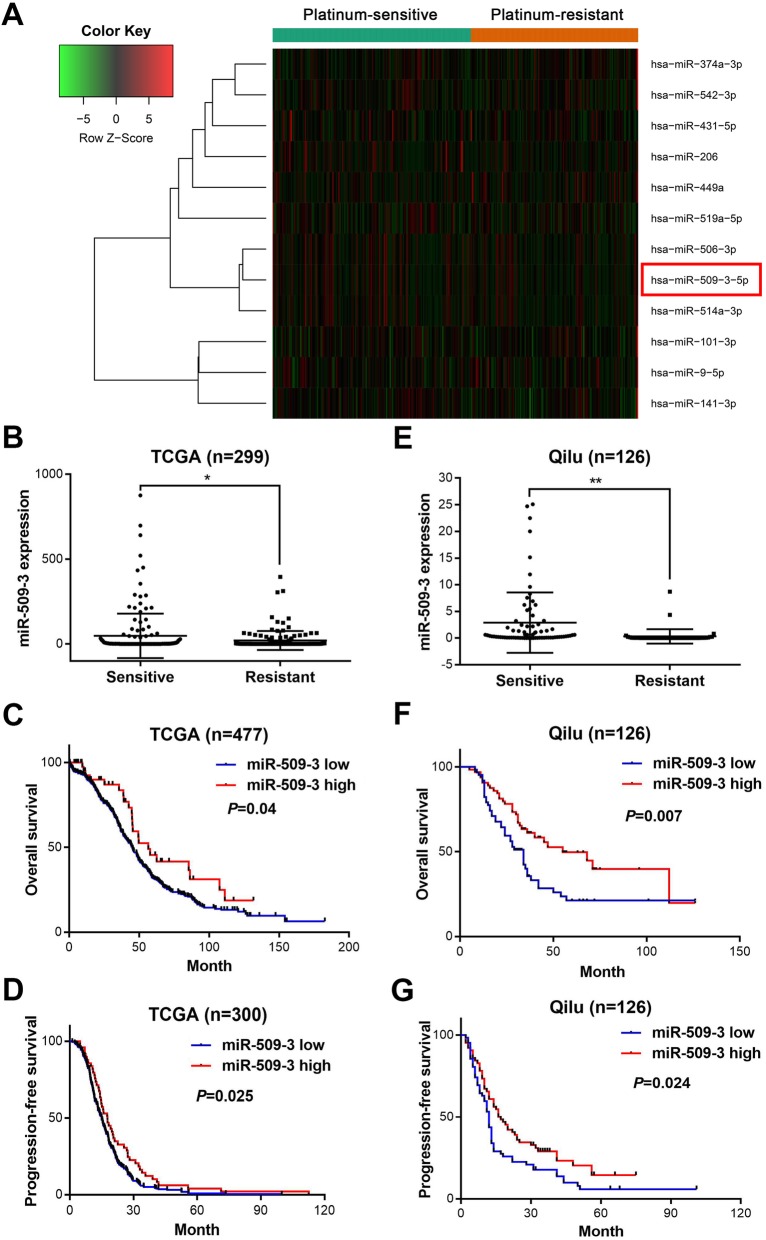
MiR-509-3 is significantly increased in P-sen group and indicated better prognosis. **a** The TCGA cohort was divided into two groups according to the clinical dataset: platinum-sensitive group (*n* = 159) and platinum-resistant group (*n* = 140). Heat map was drawn by DEGseq R package and illustrated the six top increased miRNAs and 6 top decreased miRNAs between two groups. **b** MiR-509-3 expression is elevated in platinum-sensitive group compared with platinum-resistant group in TCGA cohort (*P* < 0.05). **c** Overall survival (OS) and **d** progression-free survival (PFS) in survival curves for TCGA cohort with miR-509-3 low expression or high expression (high vs low miR-509-3 expression: OS, hazard ratio = 0.644, 95% confidence interval (CI) = 0.48 to 0.99 vs. hazard ratio = 1.551, 95% CI = 1.008 to 2.083, *P* < 0.04; PFS, hazard ratio = 0.708, 95% CI = 0.546 to 0.956 vs. hazard ratio = 1.412, 95% CI = 1.045 to 1.829, *P* < 0.02). **e** Relative miR-509-3 expression level was detected by RT-qPCR in 126 HGSOC patients from Qilu hospital. The platinum-sensitive group exhibited higher miR-509-3 expression level than platinum-resistant group (*P* < 0.01). **f** OS and **g** PFS in Qilu hospital cohort which was stratified into miR-509-3 low and high expression group whose cutoff value was defined by the medium (high vs. low miR-509-3 expression: OS, hazard ratio = 0.550, 95% CI = 0.334 to 0.830 vs. hazard ratio = 1.817, 95% CI = 1.205 to 2.987, *P* = 0.007; PFS, hazard ratio = 0.657, 95% CI = 0.432 to 0.929 vs. hazard ratio = 1.522, 95% CI = 1.076 to 2.314, *P* = 0.024)

According to the selection criteria defined in method, top six upregulated and six downregulated miRNAs in TCGA cohort were obtained and showed in the heat map (Fig. 1a and Additional file 6: Table S1). Out of the six downregulated miRNAs, miR-506 and miR-9 had been previously researched [19, 20]. The expression difference of miR-509-3 was further evaluated in the two groups by *t* test and result showed P-sen group expressed miR-509-3 in a significantly higher level than P-res group (*P* = 0.02, Fig. 1b). Elevated miR-509-3 expression indicated longer progression-free survival (PFS) and overall survival (OS) in Kaplan-Meier survival analysis (high vs. low miR-509-3 expression: OS, hazard ratio = 0.644, 95% confidence interval (CI) = 0.48 to 0.99 vs. hazard ratio = 1.551, 95% CI = 1.008 to 2.083, *P* = 0.04; PFS, hazard ratio = 0.708, 95% CI = 0.546 to 0.956 vs. hazard ratio = 1.412, 95% CI = 1.045 to 1.829, *P* = 0.02)(Fig. 1c, d). Furthermore, we performed a Cox regression analysis adjusting for FIGO stage, residual tumor size, and age at diagnosis and confirmed that miR-509-3 expression level was an independent prognostic factor in ovarian cancer (high vs. low miR-509-3 expression: OS, hazard ratio = 0.586, 95% CI = 0.371 to 0.926, *P* = 0.022; PFS, hazard ratio = 0.635, 95% CI = 0.452 to 0.892, *P* = 0.009) (Additional file 7: Table S2).

Consistent with the TCGA cohort dataset, the relationship of miR-509-3 expression level and patients’ platinum-response or prognosis were also explored in Qilu hospital cohort, which contained 126 HGSOC samples with complete clinical data (Additional file 8: Table S3). The expression of miR-509-3 in the P-sen group (*n* = 76) was also significantly higher than that of P-res group (*n* = 50) (*P* = 0.002) (Fig. 1e). And we selected the median miR-509-3 level as the cutoff value (ΔCT = 14.73) which divided the patients into high miR-509-3 group (*n* = 64) and low miR-509-3 group (*n* = 62). As for PFS or OS, high miR-509-3 group possessed significantly better prognosis than low miR-509-3 group (high vs. low miR-509-3 expression: OS, hazard ratio = 0.550, 95% confidence interval (CI) = 0.334 to 0.830 vs. hazard ratio = 1.817, 95% CI = 1.205 to 2.987, *P* = 0.007; PFS, hazard ratio = 0.657, 95% CI = 0.432 to 0.929 vs. hazard ratio = 1.522, 95% CI = 1.076 to 2.314, *P* = 0.024) (Fig. 1f, g). The Chi-squared test showed that miR-509-3 expression was correlated with platinum response (*P* < 0.0001) but not with other clinical features (Additional file 9: Table S4).

### MiR-509-3 inhibits the proliferation of ovarian cancer cells

**Fig. 2:**
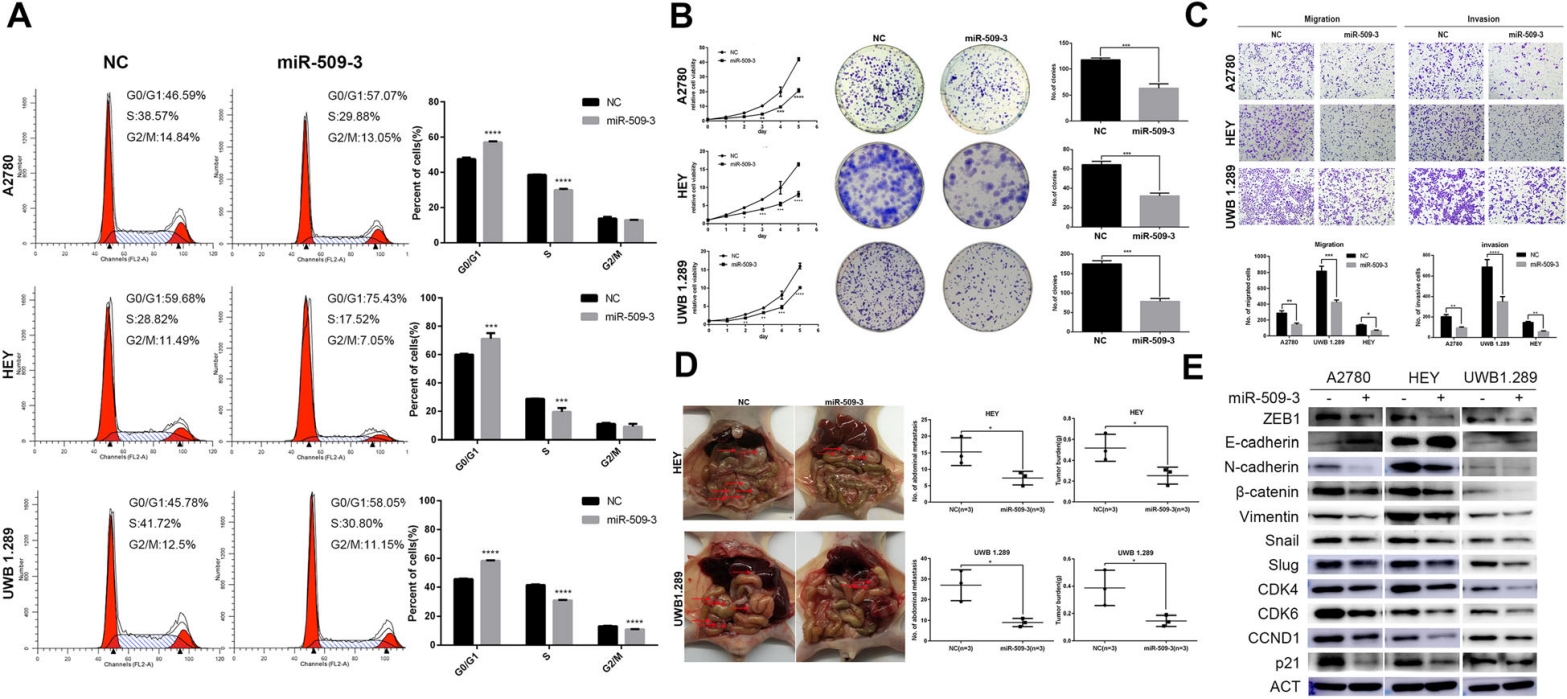
MiR-509-3 inhibits ovarian cancer cells proliferation and invasion. **a** Cell cycle analysis of A2780, HEY, and UWB1.289 when transfected with miR-509-3 mimics. miR-509-3 increased the percentage of G0/G1 phase significantly in A2780, HEY, and UWB1.289 cell lines. **b** MTT and plate clonogenic assay of three ovarian cancer cells. MiR-509-3 decreased relative cell viability at different time points (day 2 to day 5) and cell clonal number in 6-well plates. **c** Transwell assay was performed to determinate the effects of miR-509-3 overexpression on migration and invasion of A2780, HEY, and UWB1.289 cells. MiR-509-3 decreased the number of migrated cells. **d** Intraperitoneal tumor formation assay of HEY or UWB 1.289 in nude mice. MiR-509-3 impaired the number of abdominal metastasis (HEY, 15.33 ± 2.40 vs. 7.33 ± 1.20, *P* < 0.05; UWB1.289 27 ± 4.35 vs. 9 ± 1.55, *P* < 0.05) and tumor burden (HEY, 0.52 ± 0.07 vs. 0.25 ± 0.04, *P* < 0.05; UWB1.289 0.39 ± 0.07 vs. 0.15 ± 0.02, *P* < 0.05) in vivo. The red arrows indicate the tumor metastatic nodules. **e** Western blot of the EMT and cell cycle associated markers in miR-509-3 overexpressing cells. MiR-509-3 significantly downregulated ZEB1, N-cad, β-Catenin, Vimentin, Snail, and Slug but upregulated E-cad expression. G0/G1 arrest related markers CDK4, CDK6, CCND1, and p21 were also downregulated

The cell cycle analysis revealed that miR-509-3 increased the proportion of cells in G1 phase along with decline of cellular proportion in S phase (Fig. 2a). In MTT assay, relative viability of cells overexpressing miR-509-3 was significantly lower than the negative control (NC) group especially in the last 3 days (Fig. 2b). Similarly, miR-509-3 remarkably decreased the colony formation in three cell lines (*P* < 0.001) (Fig. 2b). The proteins associated with G1 phase arrest were detected by Western blot, which illustrated that miR-509-3 clearly reduced the protein expression of CDK4, CDK6, CCND1, and p21 (Fig. 2e). All the assays revealed that miR-509-3 could inhibit cell proliferation through G1 phase arrest.

### MiR-509-3 impair migration and invasion of ovarian cancer cells in vivo and in vitro

In vitro, the transwell assays with or without Matrigel Matrix showed that miR-509-3 could markedly impair the migration and invasion capacity (Fig. 2c). Equally, in vivo test, cells overexpressing miR-509-3 could form much lesser metastatic nodules in the abdominal cavity of nude mice (NC vs. miR-509-3 in HEY and UWB1.289, both *P* < 0.05) (Fig. 2d). This effect was also reflected by the epithelial-mesenchymal transition (EMT) markers. Western blot assay indicated that miR-509-3 could upregulate the epithelial marker E-cadherin and downregulated the mesenchymal markers such as ZEB1, N-cadherin, β-Catenin, Vimentin, Snail, and Slug (Fig. 2e).

### MiR-509-3 increases sensitivity to cisplatin or PARPi in ovarian cancer

**Fig. 3:**
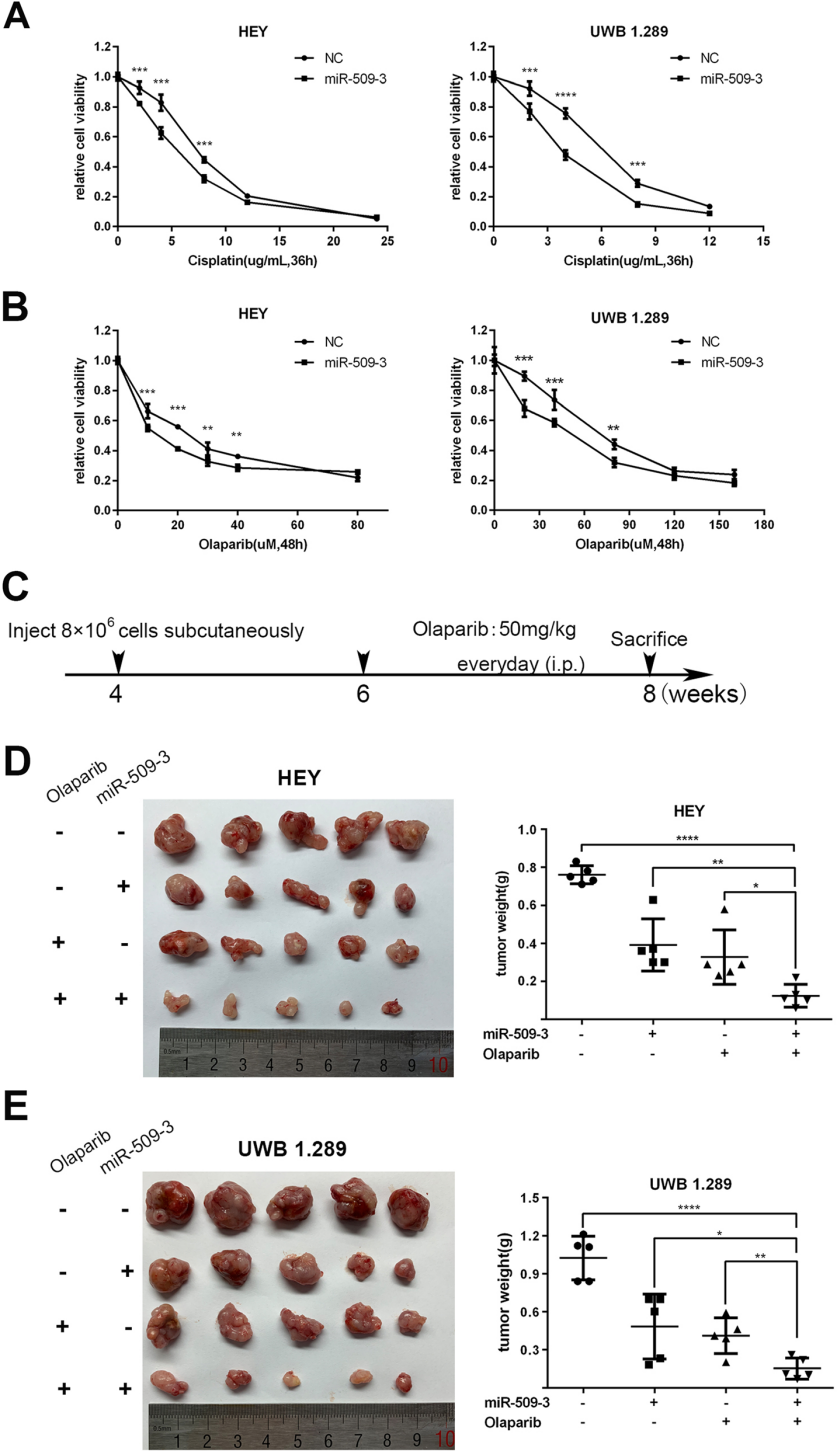
MiR-509-3 augments ovarian cancer cell sensitivity to cisplatin and Olaparib in vivo and *vitro*. **a** Relative cell viability of HEY and UWB1.289 which were exposed to different dose of cisplatin for at least 36 h and then measured by MTT method. MiR-509-3 decreased relative cell viability at various drug concentrations (HEY: 2, 4, 8 μg/mL, *P* < 0.05; UWB1.289: 2, 4, 8 μg/mL, *P* < 0.05) when compared with NC cells. **b** Relative cell viability of HEY and UWB1.289 which were treated with different dose of Olaparib (HEY: 10, 20, 30, 40, 80 μM; UWB1.289: 20, 40, 80, 120, 160 μM) for over 48 h. MiR-509-3 increased the response to Olaparib in HEY (10, 20, 30, 40 μM, *P* < 0.05) and UWB1.289 (20, 40, 80 μM, *P* < 0.05) cell lines. **c** Experimental design of tumor formation test in BALB/c nude mice. Four-week-old mice were subcutaneously injected with HEY or UWB1.289 cells (8 × 10^6^ cells per site) overexpressing miR-509-3 or corresponding NC. At the sixth week, tumor-bearing mice received intraperitoneal injection of Olaparib (50 mg/kg, once a day) for 2 weeks and then were sacrificed to harbor tumor tissues which were weighted and photographed. **d**, **e** At the eighth week of mice life, tumors harbored from each group were showed and the tumor weight was compared among four groups (data are mean ± SD, *n* = 5) in HEY and UWB1.289. Olaparib and miR-509-3 combined treatment indicated the lowest tumor weight (g) among four subgroups (HEY, 0.76 ± 0.05, 0.39 ± 0.14, 0.33 ± 0.14, 0.12 ± 0.06; UWB1.289, 1.02 ± 0.17, 0.48 ± 0.26, 0.41 ± 0.14, 0.15 ± 0.008)

Stably transfected cells overexpressing miR-509-3 and NC were exposed to cisplatin at different concentrations for 36 h. MTT assay revealed that cisplatin treatment impaired ovarian cancer cell viability in a concentration-dependent manner. And at various final concentrations, the relative cell viability of cells with miR-509-3 overexpression declined much more rapidly than the NC group (Fig. 3a).

Platinum can trigger cell apoptosis by forming intra- or inter-strand adducts with DNA which causes DNA DSBs and activate DNA damage repair pathway, especially HR repair. As a result, cells with HR repair defect are more sensitive to platinum-based therapy through synthetic lethality [21], the mechanism of which is similar to PARPi [22]. As per our expectation, cells with miR-509-3 overexpression exhibited much lower relative cell viability than NC group at the same treatment concentration (Fig. 3b).

The experiment in vivo (Fig. 3c) affirmed that both Olaparib and miR-509-3 treatment alone could significantly suppress the tumor growth while the combination of them indicated the lowest tumor burden in four subgroups (mean tumor weight of four subgroups: HEY, 0.76 ± 0.05, 0.39 ± 0.14, 0.33 ± 0.14, 0.12 ± 0.06; UWB1.289; 1.02 ± 0.17, 0.48 ± 0.26, 0.41 ± 0.14, 0.15 ± 0.008) (Fig. 3d, e).

### MiR-509-3 directly targets HMGA2 and RAD51

**Fig. 4:**
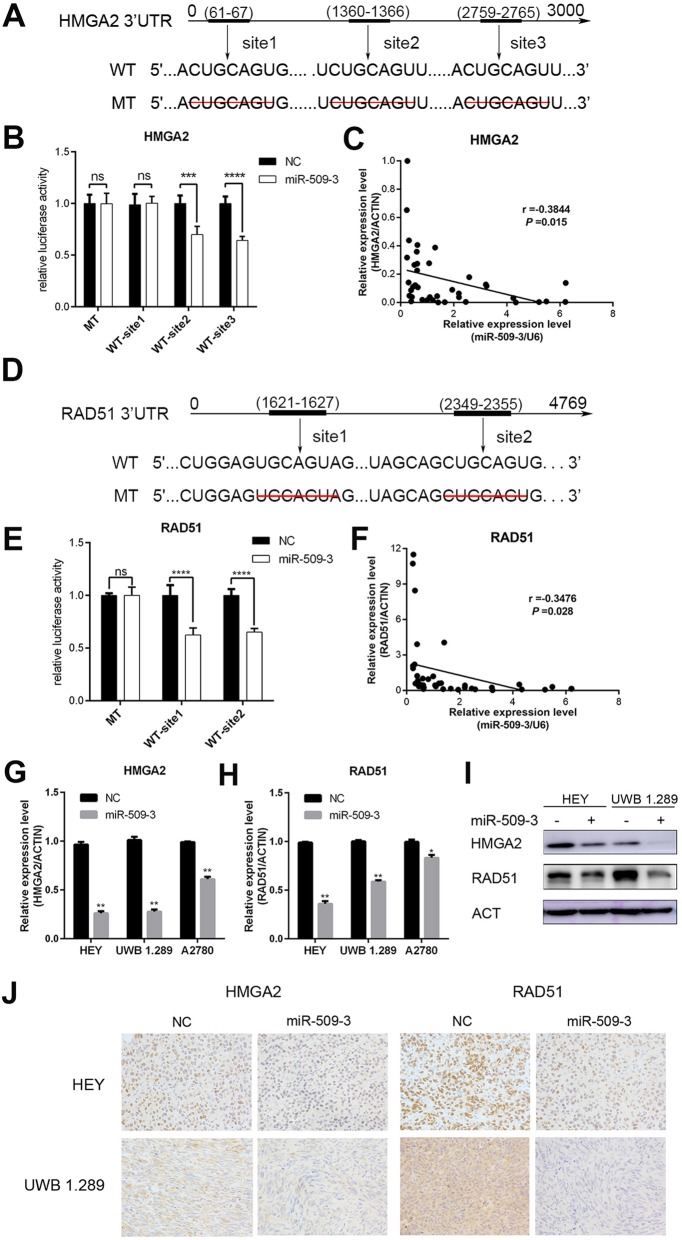
HMGA2 and RAD51 are direct targets of miR-509-3. **a** Putative binding sites in the HMGA2 3’UTR. There were three putative miR-509-3 binding sites and binding sequence was deleted as mutated type, which were cloned into pmirGLO vector respectively. **b** HEK293T cell was used in the Dual-Glo luciferase assay system. The luciferase activity of pmirGLO plasmid with wild-type (WT) HMGA2 3′UTR is weaker than that with mutant-type (MT) in HEK293T cell (site2, *P* < 0.001; site3, *P* < 0.0001). **c** Correlation between miR-509-3 and HMGA2. Relative HMGA2 mRNA level was negatively correlated with miR-509-3 level in RT-qPCR assay of tumor samples (Pearson *r* = − 0.3844, *P* = 0.015). **d**–**f** Exhibited the similar research toward RAD51 and concluded that miR-509-3 also directly targeted RAD51. **g**, **h** HMGA2 and RAD51 mRNA level in miR-509-3 overexpressing cells. Relative mRNA expressions were downregulated when cells were transfected with miR-509-3 mimics in A2780, HEY and UWB1.289. **i** Western blot assay demonstrated that miR-509-3 remarkably reduced protein expression level of HMGA2 and RAD51 in HEY and UWB1.289. **h** IHC representative image of subcutaneous tumors. The tumor tissues were fixed by formalin and cut into 4-μm-thick sections to be incubated by HMGA2 or RAD51 primary antibody. The expression of HMGA2 and RAD51 were both reduced by miR-509-3 in IHC images of HEY and UWB1.289

We predicted the targets of miR-509-3 from public available databases such as TargetScan 7.1 and miRDB and uncovered two important putative target genes, HMGA2 and RAD51. There were three potential miR-509-3 binding sites in the 3′UTR of HMGA2 (Fig. 4a). These segments of 3′UTR containing binding sites and the corresponding mutant counterparts were separately cloned into pmirGLO reporter vectors and transfected into HEK 293 T cells to detect the luciferase value differences. We confirmed that miR-509-3 overexpression markedly reduced the luciferase activity in cells transfected with wild-type site2 and site3 of 3′UTR of HMGA2 but not in cells with mutant sites in HEK 293 T cell (site2, *P* < 0.001; Site3, *P* < 0.0001) (Fig. 4b), which suggested that miR-509-3 could directly bind to site2 and site3 of HMGA2’s 3′UTR. Furthermore, we measured the relative expression levels of miR-509-3 and HMGA2 in FFPE samples of ovarian cancer patients and concluded that HMGA2 level was negatively correlated with miR-509-3 (Pearson *r* = − 0.3844, *P* = 0.01) (Fig. 4c). When it comes to RAD51, the luciferase activity was diminished in the HEK 293 T cell transfected with both two wild-type sequences of 3′UTRs compared with mutant sites which signified direct binding of miR-509-3 to RAD51 3′UTR (site1and site2, *P* < 0.0001)(Fig. 4d, e). Similarly, mRNA level of RAD51 was also negatively correlated with miR-509-3 (Pearson *r* = − 0.3476, *P* = 0.03) (Fig. 4f).

The mRNA and protein expression levels in miR-509-3 overexpressing cells and NC cells were assessed by RT-qPCR and Western blot respectively and we found that miR-509-3 could significantly downregulate the mRNA and protein levels of HMGA2 and RAD51 in ovarian cancer cells (Fig. 4g–i). The HMGA2 and RAD51 IHC staining showed consistent trend (Fig. 4j). Collectively, these data illustrated that miR-509-3 could downregulate the expression of HMGA2 and RAD51 by directly targeting their 3′UTRs.

### MiR-509-3 mediates Olaparib sensitization by downregulating RAD51 and impairing HMGA2-ATM axis

**Fig. 5:**
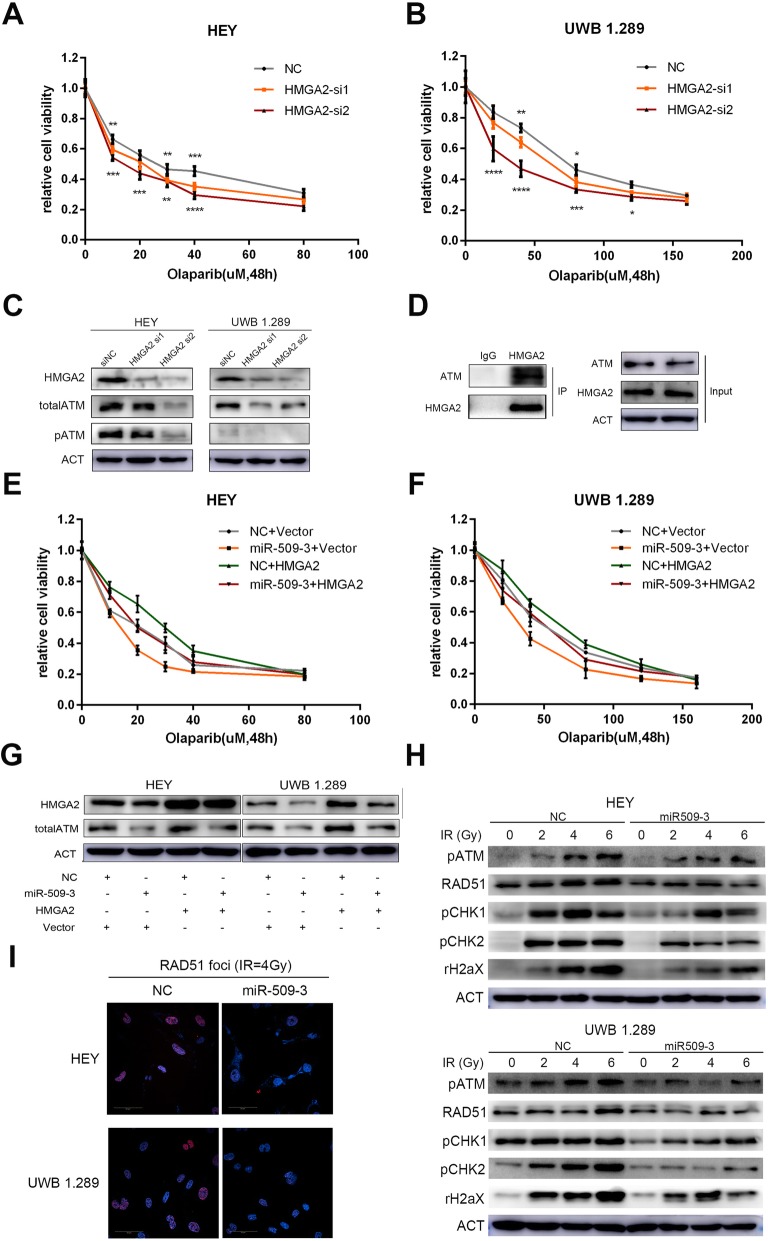
MiR-509-3 increases sensitivity to Olaparib by downregulating RAD51 and impairing HMGA2-ATM axis in ovarian cancer cell. **a**, **b** Relative cell viability of HEY and UWB1.289 which were transfected with two HMGA2 siRNAs and then treated with Olaparib for 48 h in accordance with the previous concentration gradient. The viability curve illustrated that HMGA2 downregulation enhanced the cell response to Olaparib significantly. **c** Western blot assay confirmed the interference efficiency of two HMGA2 siRNAs. And the total ATM and activated ATM protein level were both decreased along with the down-regulation of HMGA2. **d** Co-immunoprecipitation of HMGA2 and ATM protein. HEY cells were lysed and mixed with rabbit anti-human HMGA2 polyclonal antibody or normal rabbit IgG. Anti-HMGA2 antibody could co-immunoprecipitate ATM and HMGA2 while immunoglobin G (IgG) had no effect. **e**, **f** Rescue impact of HMGA2 on the response to Olaparib. Transfection of HMGA2 cDNA (in pEnter vector) could abrogate the miR-509-3-induced Olaparib sensitivity to Olaparib in HEY and UWB1.289 cell lines. The cell viability curves of different groups were drawn by four colors. **g** Protein level of four rescue groups. For the four treatment groups, transfection of HMGA2 cDNA could indeed upregulate the HMGA2 protein level and total ATM expression down-regulation was rescued by up-regulation of HMGA2 in miR-509-3 overexpressing cells. **h** HEY and UWB1.289 cells with miR-509-3 or NC were exposed to irradiation at different dose (0, 2, 4, 6 Gy) to induce DNA damage and repair system. MiR-509-3 overexpressing group significantly exhibited a lower protein level of ATM-CHK1/2 pathway (phospho-ATM, phospho-CHK1, phospho-CHK2 and phospho-H2AX.) and RAD51. **i** IF images of RAD51 foci in HEY and UWB1.289. Cells were treated with irradiation of 4Gy and 4 h later, RAD51 functional nuclear foci formation was detected by IF photographed and was found to be remarkably reduced by miR-509-3. Nuclei were stained with DAPI (blue)

HMGA2 has been confirmed to interact with multiple DNA damage repair factors such as ATR, ATM, and ERCC [23–27] to potentially mediate response to PARPi. Considering the key role of ATM (ataxia telangiectasia mutated kinase) in DSB repair, we hypothesized that HMGA2 could regulate ATM to influence the sensitivity to PARPi in ovarian cancer cell. Two siRNA sequences of HMGA2 were transiently transfected into cells and the interference efficiency was confirmed. Downregulation of HMGA2 could increase the sensitivity to Olaparib in ovarian cancer cells (Fig. 5a, b). Protein expression level of total ATM and functional phospho-ATM were also detected to trend downward (Fig. 5c). ATM was also co-immunoprecipitated by anti-HMGA2 antibody (Fig. 5d).

To further explore whether the effect of miR-509-3 was achieved through HMGA2, we designed the rescue experiment by transiently transfecting HMGA2 cDNA (in pEnter) without 3′UTR or blank pEnter vector in miR-509-3 overexpressing cells or corresponding NC cells and established four subgroups. The relative cell viability of the four subgroups when exposed to gradiently diluted Olaparib was also measured and the result revealed that the overexpression of HMGA2 could partly abrogate the increased sensitivity to Olaparib conferred by miR-509-3 overexpression (Fig. 5e, f). The transwell assay showed that upregulation of HMGA2 could partially counteract miR-509-3-mediated inhibition on invasion of ovarian cancer cells, which indicated that miR-509-3 inhibited invasion by targeting HMGA2 (Additional file 1: Figure S1). The analogous rescue experiment was also performed by transfecting RAD51 cDNA, which confirmed that RAD51 overexpression could also reverse the sensitization effect of miR-509-3 (Additional file 2: Figure S2). Furthermore, the total ATM expression was also depressed by miR-509-3, whereas rescued by transfection of HMGA2 cDNA (Fig. 5g). Additionally, we treated the cells with different doses of irradiation to induce DNA damage and observed the dynamic change of activated ATM pathway. The Western blot assay illustrated that miR-509-3 could impair ATM-CHK1/2 pathway activation when cells were challenged with external stressors (Fig. 5h). And the RAD51 fluorescence foci formation was impaired by miR-509-3 overexpression in irradiation exposure (Fig. 5i). Above all, these results suggested to us that miR-509-3 mediated Olaparib sensitization by directly impairing HMGA2-ATM axis as well as downregulating RAD51 in ovarian cancer.

### Establishment of PDX models and their genomic characterizations

To further investigate the exact therapeutic effect of Olaparib and miR-509-3 in ovarian cancer tissue derived from clinical patients, we established proven PDX model containing 9 HGSOC cases.

**Fig. 6:**
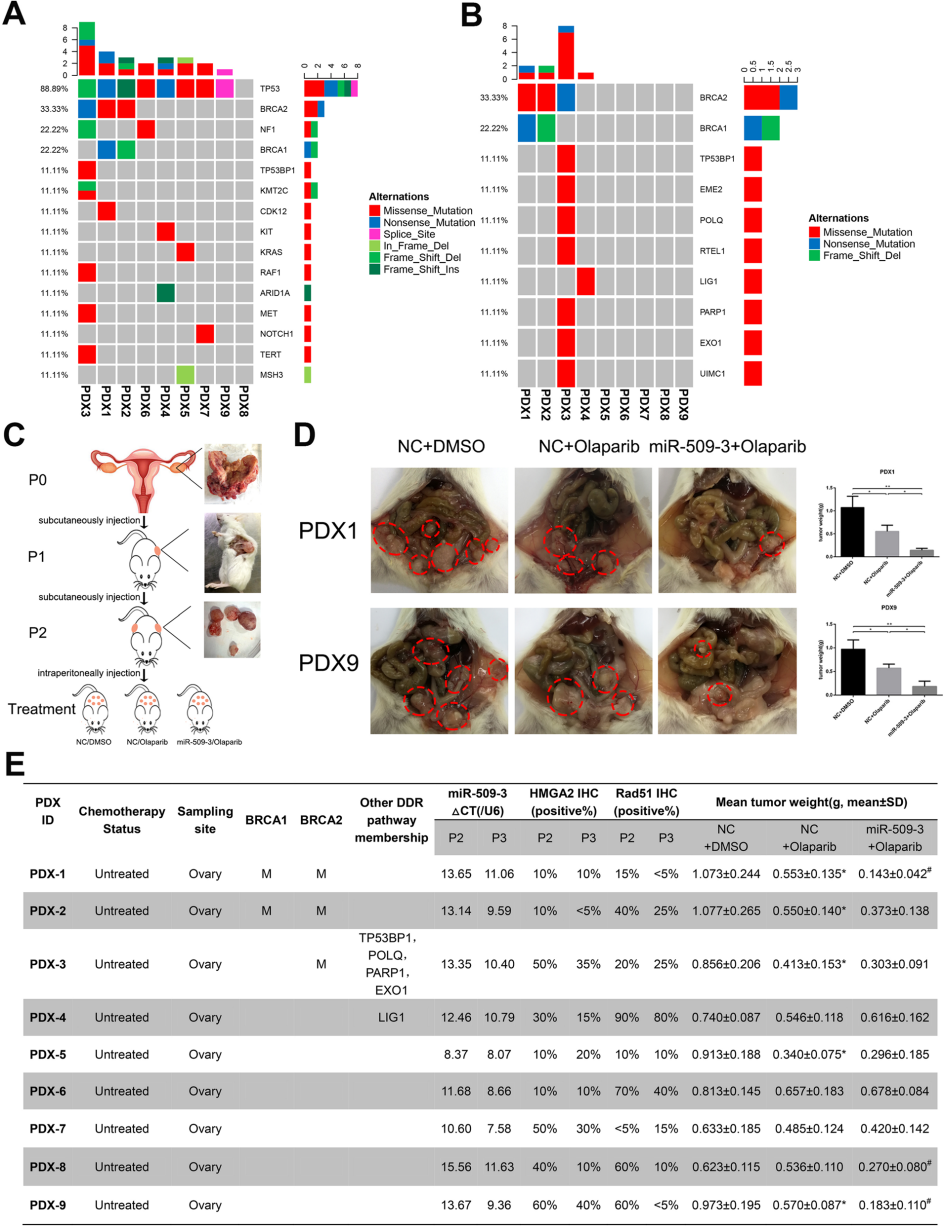
Genomic characterizations of the established PDX cases and Olaparib or miR-509-3 treatment responses in PDX models. **a** Ovarian cancer-driven gene mutation landscape of cases. Tumor and paired normal tissues of nine enrolled patients were sequenced by WES and 157 genes associated with ovarian and/or breast cancer susceptibility or tumorigenesis were annotated. The landscape of 9 PDX cases revealed the chief mutated genes in these HGSOC patients. The TP53 mutation rate is 88.89% (8/9). **b** HR pathway gene mutation landscape of cases. The landscape demonstrated that 44.44% (4/9) cases harbored HR gene mutation in which BRCA1/2 accounted for 75% (3/4). **c** The procedure of PDX model establishment and application in this study. P0 tumor tissues were obtained from gynecological surgery and implanted subcutaneously as P1. In the treatment groups (P3), AAV-miR-509-3 or AAV-NC were injected intraperitoneally twice within one week and then treated with Olaparib (50 mg/kg) once a day for 2 weeks. **d** Typical cases of miR-509-3 sensitization. PDX1 and PDX9 were representative cases in which miR-509-3 could enhance the sensitivity to Olaparib (mean tumor weights of NC + DMSO, NC + Olaparib, or miR-509-3 + Olaparib group respectively: PDX1, 1.073 ± 0.244, 0.553 ± 0.135, 0.143 ± 0.042; PDX9, 0.973 ± 0.195, 0.570 ± 0.087, 0.183 ± 0.110). Dotted red lines circled the tumor site in the abdomen of NCG mice. **e** The summarized list of detailed characteristics of each PDX cases including treatment status, sample origins, HR gene mutation status, endogenic miR-509-3 expression, HMGA2-positive rate, RAD51-positive rate, and mean tumor weight of three treatment groups (showed as means ± SD). In this figure, label “*” means this case is sensitive to Olaparib. Label “#” means miR-509-3 has sensitizing effect

Considering the possibility of variant therapeutic response on account of the genetic alterations in PDX models, we queried the WES data from the parent tumors (P0) for somatic mutations in ovarian cancer-driven genes. Consequently, 157 genes implicated in ovarian and/or breast cancer susceptibility or tumorigenesis were analyzed [28]. Not surprisingly, 88.89% (8/9) of HGSOC patients harbored a mutation in TP53 except PDX8 (Fig. 6a) but all cases exhibited a high expression level of TP53 in IHC staining (Additional file 3: Figure S3). Apart from TP53, another ovarian-driven gene mutation such as BRCA1/2, NF1, CDK12, KRAS, etc. were also detected occasionally in some patients (Fig. 6a). Furthermore, DDR gene list including 276 genes was compiled referring to Theo A [29]. and the alteration analysis illustrated that the HRR gene mutation was observed in 44.44% (4/9) patients, in which BRCA1/2 mutation accounted for 75% (3/4) (Fig. 6b). The detailed ovarian cancer-driven and DDR genes mutation information of the nine patients is provided in Additional file 10: Table S5 and Additional file 3: Figure S3.

The systematic experimental process is displayed in Fig. 6c and 64% (9/14) of patients’ samples could be stably passaged and used in this assay. The HE and TP53 features from P0 to P3 of these nine used PDX cases were exhibited in Additional file 4: Figure S4.

### Therapeutic effect of Olaparib and/or miR-509-3 in PDX models

To assess the therapeutic effects of Olaparib and/or miR-509-3, the mean tumor burden of each treatment group in 9 PDX cases was weighted and reported in Fig. 6d, e. The RAD51 or HMGA2 expression of P2 (not treated) and P3 (treated with Olaparib and miR-509-3) were stained by IHC and the positive cell ratio was measured. As showed in Fig. 6e, the RAD51-positive ratio of five PDX samples was less than 50% and that of the other four cases exceeded 50%. After treatment of miR-509-3, five cases presented evident decline in RAD51 positive ratio. And the HMGA2-positive rates were significantly decreased in 66.7% (6/9) cases when treated with miR-509-3 (Fig. 6e and Additional file 4: Figure S4).

We divided nine PDX cases into high-miR-509-3 group (*n* = 8) and low-miR-509-3 group (*n* = 1, PDX8) according to the cutoff value in 126 Qilu hospital patients. Further, 62.5% (5/8) of high miR-509-3 cases were sensitive to Olaparib while no low miR-509-3 case responded to Olaparib.

In the four HR gene mutation cases, three of them (PDX1, PDX2, PDX3) exhibited statistically significant decrease in tumor burden when treated with Olaparib (*P* < 0.05 for all cases) and unsurprisingly all harbored BRCA1/2 mutation while the PDX4 who carried LIG1 mutation did not respond to Olaparib. However, among the five HR wild-type cases, only two of them (PDX5 and PDX9) responded effectively to Olaparib treatment. Furthermore, we observed that in patients possessing a low primary RAD51 positive rate (less than 50%), 80% (4/5) of them were sensitive to Olaparib treatment (*P* < 0.05) while the rate dropped to 25% (1/4) in high RAD51 positive rate (over 50%) cases. In summation, the overall effective rate of Olaparib in the nine PDX cases was 55.6% (5/9) and the patients with HR gene mutation or low RAD51-positive rate were more sensitive to Olaparib. As for HMGA2, we observed that in six cases whose HMGA2 was decreased after AAV-miR-509-3 injection, 66.7% (4/6) of them were sensitive to Olaparib and/or miR-509-3 therapy (Fig. 6e and Additional file 5: Figure S5).

When taking miR-509-3 treatment into comparison, we were glad to discover that in the PDX1 and PDX9 cases, combination of Olaparib and miR-509-3 could much more significantly decrease the tumor burden than Olaparib monotherapy (mean tumor weights of NC + DMSO, NC + Olaparib, or miR-509-3 + Olaparib group respectively: PDX1, 1.073 ± 0.244, 0.553 ± 0.135, 0.143 ± 0.042; PDX9, 0.973 ± 0.195, 0.570 ± 0.087, 0.183 ± 0.110) (Fig. 6d). More surprisingly, miR-509-3 could reverse the resistance to Olaparib in PDX8 (three groups: 0.623 ± 0.115, 0.536 ± 0.110, 0.270 ± 0.080) and all of these three cases exhibited decreases in RAD51 positive rate after being treated with miR-509-3. However, for the other six PDX cases, miR-509-3 did not remarkably improve the treatment outcome of Olaparib. All these mean tumor weights statistics and representative tumor graphs are presented in Fig. 6d, e and Additional file 5: Figure S5.

## Discussion

Although BRCA1 and BRCA2 (BRCA1/2) mutation status were regarded as an important indicator in the application of PARPi in ovarian cancer patients [10], there were still 40–70% of patients not responding expectedly to Olaparib as a result of drug resistance through multiple mechanisms such as restoration of BRCA1 or BRCA2 protein functionality by secondary mutations, BRCA1/2 promoter methylation reversion, BRCA1/2 hypomorph overexpression, PARP1 expression deficiency, drug efflux, or acquisition of new mutations in other DDR genes [30–33]. That is to say simply that BRCA1/2 mutation detection cannot be an ideal indicator for predicting PARPi sensitivity. Aiming at this present condition, scholars have developed better HRD detection methods to predict the sensitivity to PARPi or prognosis of ovarian cancer patient, in which the HRR Gene Panel and HRD Genomic Scar are the most well-known. For example, a 30-gene panel was proved to have a positive impact on overall survival and platinum response [7]. A 61-gene panel was developed to distinguish BRCA-like tumors from non-BRCA-like tumors. Meanwhile, “BRCAness profile” correlates with sensitivity to platinum and PARPi and identifies a group of sporadic patients with good prognosis [34]. In view of the increasing complexity of mutation analysis of all HR pathway genes, the genomic scar analysis emerged. Genomic scar means the gain or loss of large chromosomal regions or even whole chromosomes and can be observed as copy number variations resulting from DNA damage repair failure, which is focusing on DDR pathways instead of DDR genes [35]. Genomic scar analysis, which mainly consist of “My Choice” test from Myriad Genetics or “Foundation Focus” test from Foundation Medicine, is believed to be more efficient in identifying “BRCA-like” tumors and has thus been undergoing clinical trials to discriminate between HR-proficient and HR-deficient tumors [35–37]. However, the HRD negative patients could also benefit from PARPi. A randomized, double-blind, phase 3 trial containing 553 enrolled patients revealed that Niraparib could both improved progression-free survival in the HRD-negative subgroup and HRD-positive subgroup in non-gBRCA cohort [15]. In another phase 3 clinical trial, the investigators classified that in patients with BRCA wild-type carcinomas, a benefit was also seen with rucaparib in patients with both high-LOH and low-LOH carcinomas [16]. In sight of the limitation of genomic analysis, currently, scholars have devised a platform for functionally profiling DNA repair in short-term patient-derived HGSOC organoids. Their research results indicated that a combination of genomic analysis and functional testing of DDR pathway allows for the identification of DNA repair inhibitor including Olaparib [38]. In a pan-cancer research, the somatic alterations of 33 cancer types was systematically analyzed to provide a comprehensive view of DDR deficiency, which defined a “core DDR” gene set of 80 genes from 276 genes encompassing all major DNA repair pathways including HR core gene [29]. Our DDR mutation analysis of nine PDX cases was based on this study. In the HR positive patients, 75% (3/4) of them (PDX1, PDX2, and PDX3) harboring BRCA1/2 or other HR core gene mutation could effectively response to Olaparib treatment. Additionally, the only case (PDX4) who carried LIG1 mutation (a non-HR core gene) exhibited resistance to Olaparib. Therefore, we proposed that HR core gene analysis could be a potentially effective means for Olaparib response prediction in clinical application. However, this proposal requires a larger size preclinical study to be confirmed.

RAD51, a kind of DNA recombinase, is a crucial downstream component in the HR repair pathway. When cells are exposed to stressors (especially irradiation), RAD51 is relocalized within the nucleus to form distinct foci, which are thought to represent assemblies of HR repair proteins at these sites [39]. Therefore, targeting RAD51 could confer cells to be defective in HR repair and the sensitivity to DNA-damaging drugs including platinum and PARPi [32, 40]. More importantly, several recent studies have revealed that functional assay of RAD51 nuclear foci can be a surrogate marker for HR repair functionality and thus be used to predict the response to PARPi in vitro [41]. Cruz et al. found that elevated RAD51 nuclear foci were the only common feature in PDX and patient samples with primary or acquired PARPi resistance [42]. Meanwhile, the RAD51 score could more predictively discriminate PARPi sensitivity between PARPi resistance in breast cancer PDXs than the conventional genomic test [43]. However, in our research, 20% (1/5) PDX cases with low primary RAD51 positive rate were not sensitive to Olaparib while 25% (1/4) with high rate actually responded well to Olaparib, and the Olaparib-sensitive PDX cases all showed low RAD51 expression rate in P3 (after treatment) group. This exception may be rationalized by the fact that RAD51 status would be better represented when cells were challenged with stressors such as irradiation. Similarly, in another study using patient-derived organoids, researchers similarly demonstrated that Olaparib sensitivity correlated with the absence of RAD51 foci formation after irradiation exposure [38]. Therefore, we were convinced that functional detection of RAD51 (often treated with irradiation, etc.) is still a promising method in predicting PARPi response.

MiRNAs have already been hopeful tools and targets for novel therapeutic approaches and showed application prospects in preclinical study. For example, the mimic of tumor suppressor miRNA miR-34 has been undergoing phase 1 clinical trials (NCT01829971) in cancer therapy and the antimiRs targeting miR-122 in phase 2 clinical trials (NCT01872936, NCT02031133, NCT02508090) for hepatitis [44, 45]. Furthermore, miRNAs have been proven to play a definite role in the PARPi response by regulating the HR pathway, such as miR-9, miR-506, miR-223, miR-182, miR-96, miR-622, miR-493, etc. [19, 20, 46–49]. For instance, Sun et al. reported that miR-9 mediated the downregulation of BRCA1 and impeded DNA damage repair to improve chemotherapeutic and PARPi efficacy [19]. Additionally, Srinivasan et al. found that miR-223 was a negative regulator of the NHEJ DNA repair and represented a therapeutic pathway in BRCA1-deficient cancers [49]. In this study, we explored the TCGA dataset and uncovered miRNA-509-3 as a novel miRNA which predicted a better response to platinum-based chemotherapy and longer survival in ovarian cancer patients. We determined that miR-509-3 was a definite tumor suppressor that could significantly impact ovarian cancer cell metastasis, proliferation, and cell cycle. A series of cellular functional assay illustrated that miR-509-3 enhanced sensitivity to Olaparib by directly regulating RAD51 and HMGA2-ATM axis and performing synthetic lethal effect. Consistent with the result of in vitro experiment, the PDX model tumor burden of three treatment groups illustrated that when combined with miR-509-3, Olaparib was more effective in reducing tumor burden in two PDX cases (PDX1 and PDX9) along with the decline in RAD51 positive rate. Surprisingly, in one case (PDX8), miR-509-3 could reverse the Olaparib insensitivity by downregulating RAD51 expression. What is more, we consider that miR-509-3 level is positively correlated to the sensitivity to Olaparib, which needs more samples in the future. Consequently, these provided us with a potential promising target in Olaparib treatment.

## Conclusion

In conclusion, strategies to optimize the approaches capitalizing on synthetic lethality with HR deficiency are imperative. Both HR core gene analysis and RAD51 functional detection are potentially feasible in PARPi response discriminating and predicting. And we have found a novel miRNA that directly regulated HR repair which indicated that combination with miR-509-3 may enhance the therapeutic effect of PARPi in certain cases. However, its limitation should not be ignored that more preclinical PDX cases should be enrolled into study which is our next venture in our research direction.

## Supplementary information

**Additional file 1: Figure S1**. A) and B) The impairment of invasion caused by overexpression of miR-509-3 could be rescued by introduction of HMGA2.

**Additional file 2:Figure S2** . A) and B) RAD51 overexpression could reverse the sensitizing effect of miR-509-3. C) RAD51 protein expression level in rescue groups.

**Additional file 3: Figure S3**. A) MMR mutation, B) TLS mutation, C) FA mutation and D) BER mutation landscapes of the PDX cases.

**Additional file 4: Figure S4**. HE, TP53, RAD51 and HMGA2 staining images of 9 used PDX cases.

**Additional file 5: Figure S5**. Tumor weight statistics graphs and representative tumor photographs of PDX2 to PDX8

**Additional file 6: Table S1**. Log2 fold change, *P* value and FDR of miRNAs between platinum-sensitive group and platinum-resistant group in TCGA cohort.

**Additional file 7: Table S2**. Cox regression analysis of TCGA cohort adjusting for FIGO stage, residual tumor size and age at diagnosis.

**Additional file 8: Table S3**. Detailed clinical characteristics of each enrolled HGSOC patients from Qilu hospital.

**Additional file 9: Table S4**. Correlation between miR-509-3 expression and clinical features in Qilu hospital cohort.

**Additional file 10: Table S5**. Ovarian cancer driven genes and DDR related genes mutation information of 9 PDX samples.

## Data Availability

The data used and/or analysis during the current study are available from the corresponding author on reasonable request.

## Abbreviations

3′UTR: 3′ Untranslated regions
DDR: DNA damage response
FFPE: Formalin-fixed and paraffin-embedded
HGSOC: High-grade serous ovarian carcinoma
HR: Homologous recombination
HRD: HR deficiency
IHC: Immunohistochemistry
MMR: Mismatch repair
NC: Negative control
NHEJ: Non-homologous end joining
PARPi: Poly (ADP­ribose) polymerase inhibitors
PDX: Patient-derived xenograft
RT-qPCR: Real-time quantitative PCR
siRNA: Small interfering RNA
WES: Whole-exome sequencing
