# Supplementary figures and images for: MiR-509-3 augments the synthetic lethality of PARPi by regulating HR repair in PDX model of HGSOC
Source: J Hematol Oncol. 2020 Jan 31;13:9. doi: 10.1186/s13045-020-0844-0 (PMC6995078; doi:10.1186/s13045-020-0844-0)

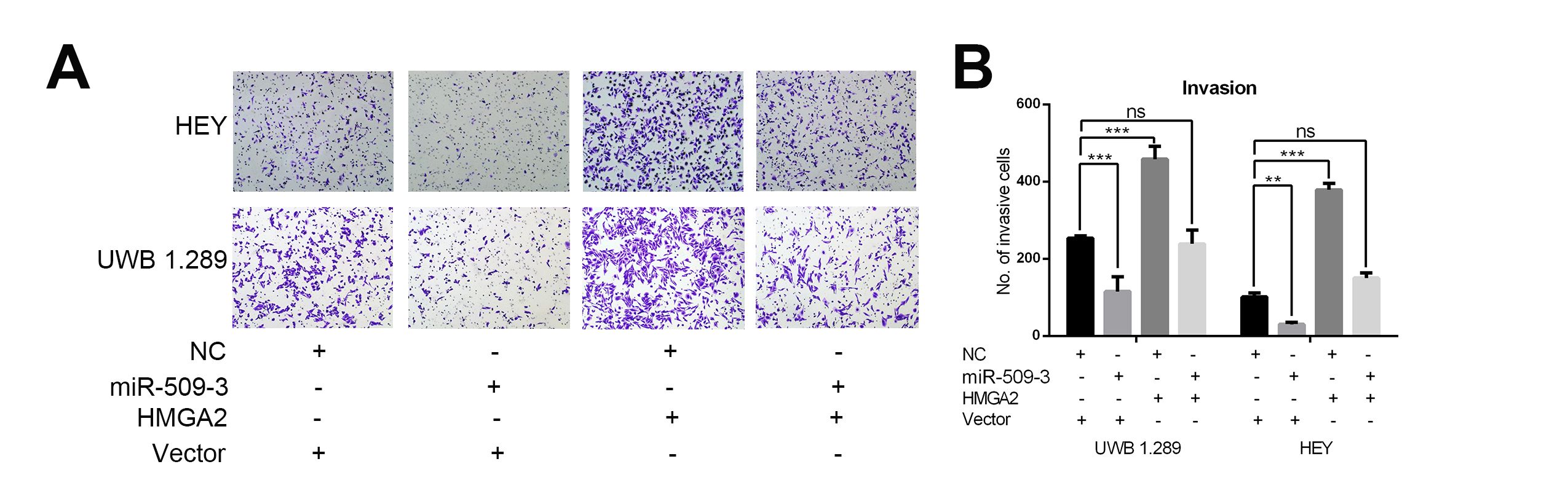

Supplement: Supplementary file 1 — Additional file 1: Figure S1. A) and B) The impairment of invasion caused by overexpression of miR-509-3 could be rescued by introduction of HMGA2. [file 13045_2020_844_MOESM1_ESM.jpg]

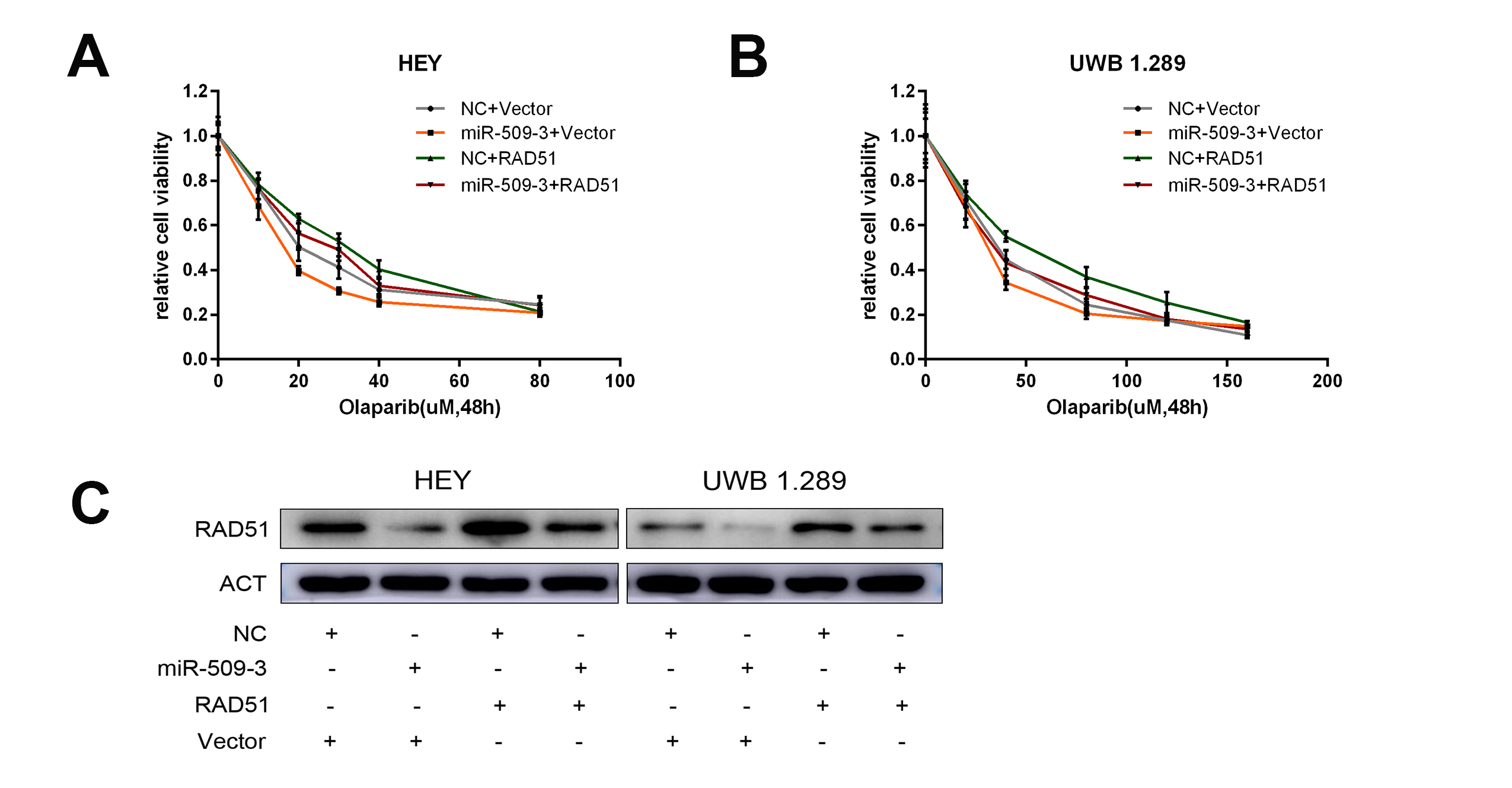

Supplement: Supplementary file 2 — Additional file 2:Figure S2 . A) and B) RAD51 overexpression could reverse the sensitizing effect of miR-509-3. C) RAD51 protein expression level in rescue groups. [file 13045_2020_844_MOESM2_ESM.jpg]

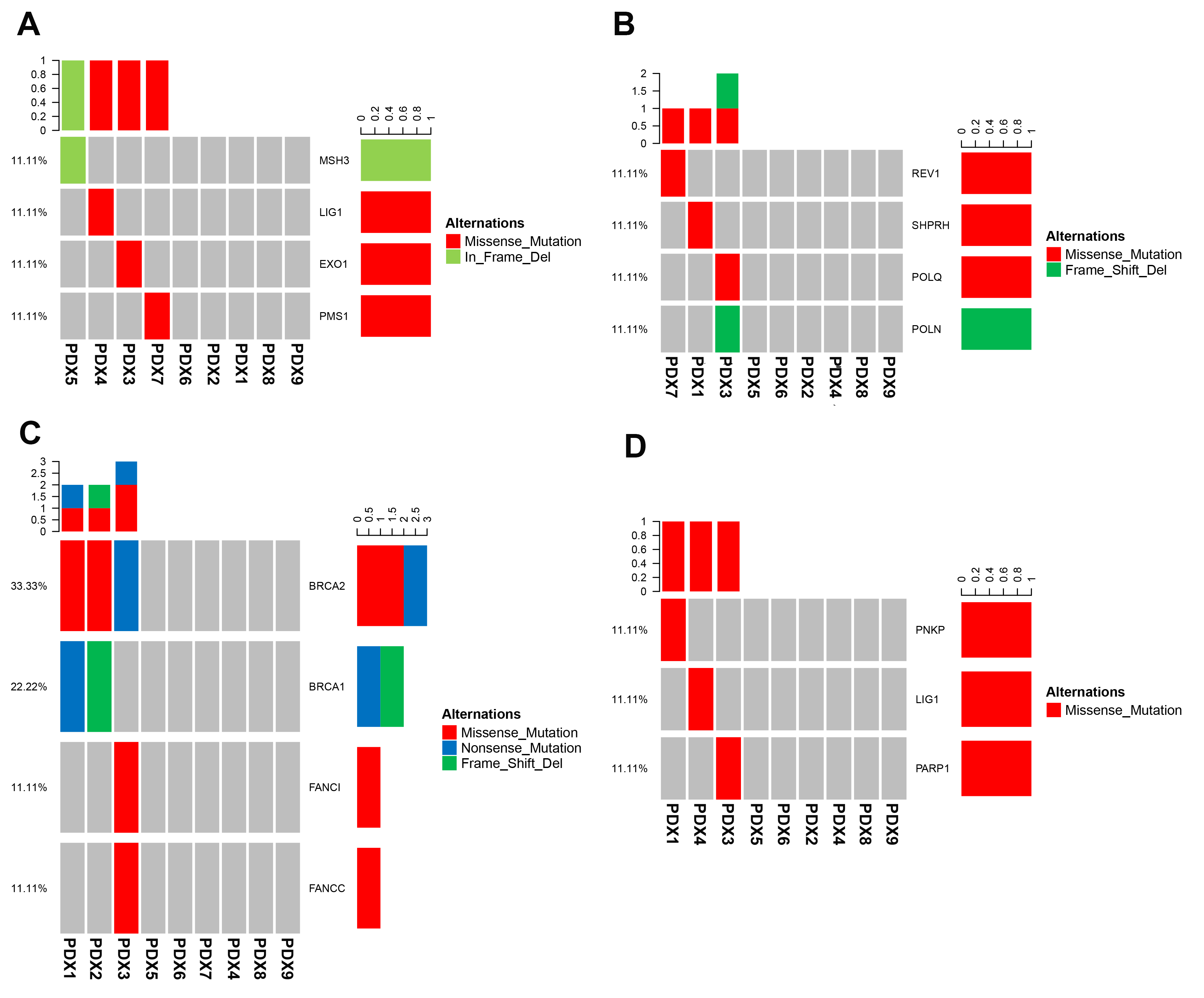

Supplement: Supplementary file 3 — Additional file 3: Figure S3. A) MMR mutation, B) TLS mutation, C) FA mutation and D) BER mutation landscapes of the PDX cases. [file 13045_2020_844_MOESM3_ESM.jpg]

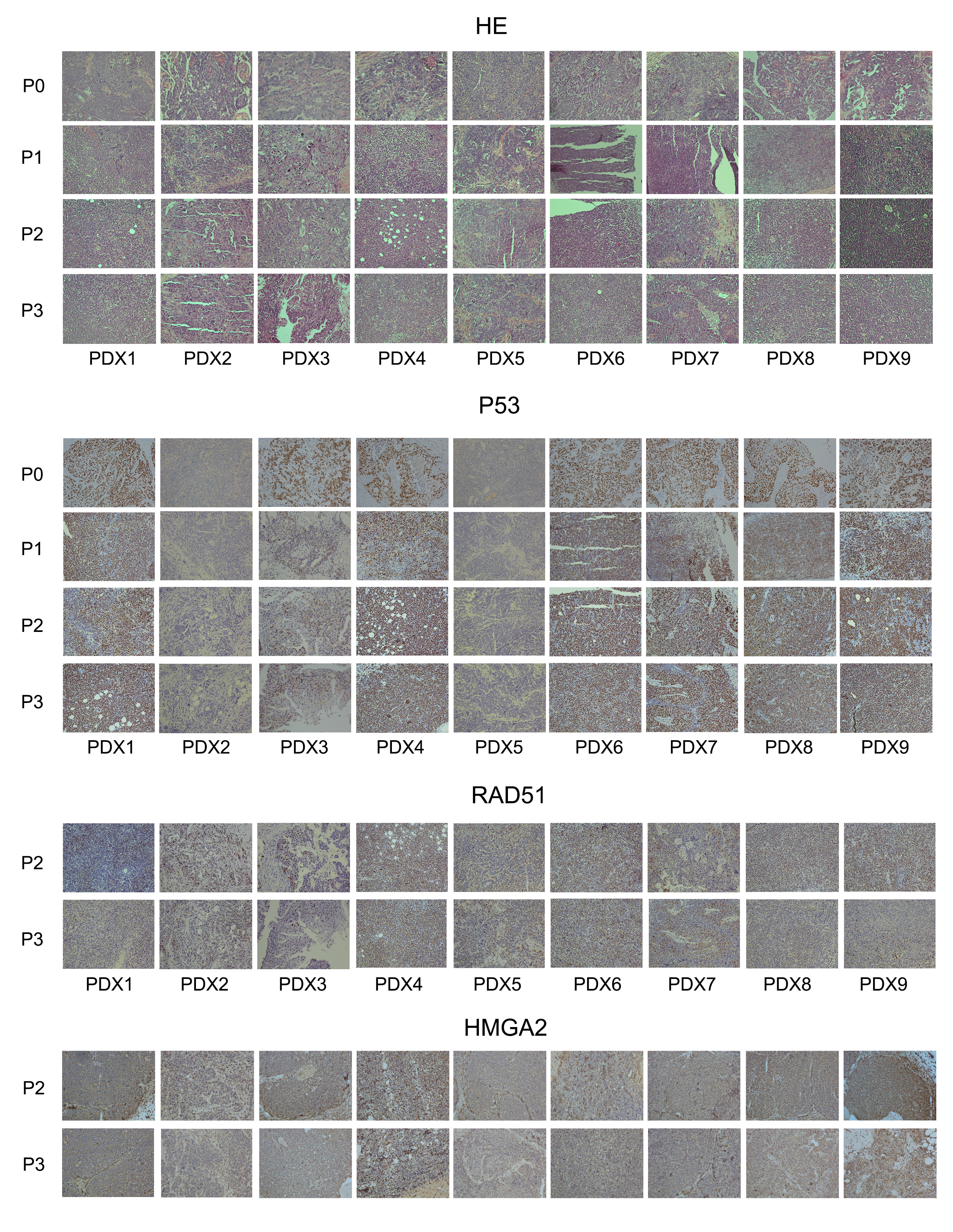

Supplement: Supplementary file 4 — Additional file 4: Figure S4. HE, TP53, RAD51 and HMGA2 staining images of 9 used PDX cases. [file 13045_2020_844_MOESM4_ESM.jpg]

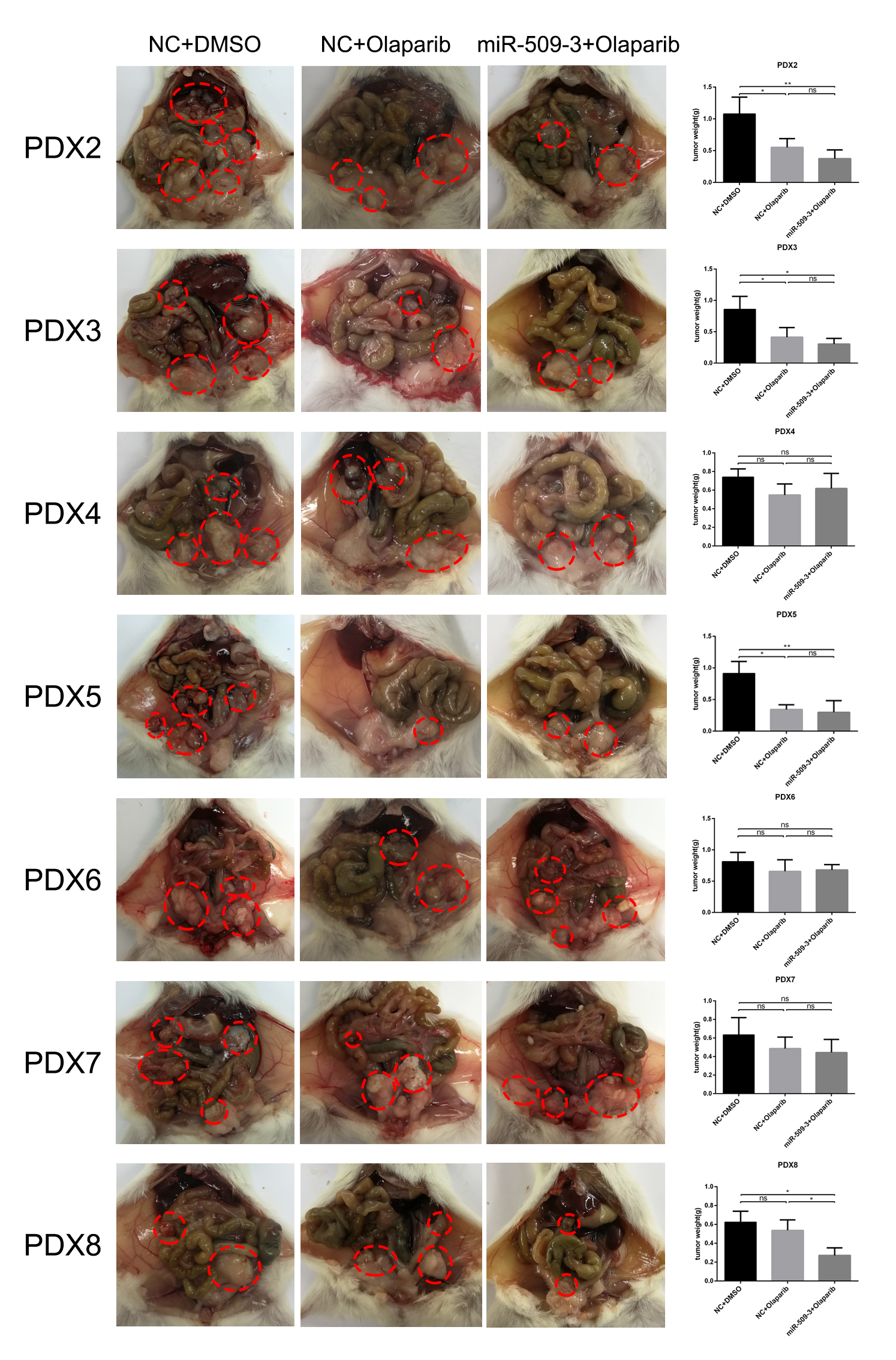

Supplement: Supplementary file 5 — Additional file 5: Figure S5. Tumor weight statistics graphs and representative tumor photographs of PDX2 to PDX8 [file 13045_2020_844_MOESM5_ESM.jpg]
