# Supplementary material for: MiR-509-3 augments the synthetic lethality of PARPi by regulating HR repair in PDX model of HGSOC
Source: J Hematol Oncol. 2020 Jan 31;13:9. doi: 10.1186/s13045-020-0844-0 (PMC6995078; doi:10.1186/s13045-020-0844-0)
Supplement: Supplementary file 7 — Additional file 7: Table S2. Cox regression analysis of TCGA cohort adjusting for FIGO stage, residual tumor size and age at diagnosis. [file 13045_2020_844_MOESM7_ESM.docx]

**Supplementary table 2. Cox regression model of TCGA cohort**

|  | OS  HR（95%CI） | *P* | PFS  HR（95%CI） *P* | |
| --- | --- | --- | --- | --- |
| miR-509-3 status  low-expression  high-expression  FIGO stage  I+II  III+IV  Tumor residual  <1mm  >1mm  Age  <60  >60 | 1  0.586(0.371-0.926)  0.381(0.157-0.927)  1  0.700(0.537-0.912)  1  0.724(0.568-0.922)  1 | 0.022  1  0.635(0.452-0.892)  0.033  0.632(0.341-1.169)  1  0.008  1  1.113(0.829-1.496)  0.009  0.805(0.621-1.043)  1 | | 0.009  0.144  0.476  0.100 |
