# Supplementary material for: MiR-509-3 augments the synthetic lethality of PARPi by regulating HR repair in PDX model of HGSOC
Source: J Hematol Oncol. 2020 Jan 31;13:9. doi: 10.1186/s13045-020-0844-0 (PMC6995078; doi:10.1186/s13045-020-0844-0)
Supplement: Supplementary file 9 — Additional file 9: Table S4. Correlation between miR-509-3 expression and clinical features in Qilu hospital cohort. [file 13045_2020_844_MOESM9_ESM.docx]

**Supplementary table 4. Correlation between miR-509-3 expression and clinical features**

| Clinical features | miR-509-3 status  Low expression High expression | | *P* |
| --- | --- | --- | --- |
| Age  <55  >55  FIGO Stage  I+II  III+IV  CA125 level  <700  >700  Platinum response  Sensitive  Resistant | 31  31  9  53  29  33  20  42 | 29  35  12  52  36  28  56  8 | 0.60  0.52  0.29  <0.0001 |
